# Belonging in Uncertain Times: An Ecological Perspective on the Identification of LOTE Majors

**DOI:** 10.3390/bs16071088

**Published:** 2026-07-02

**Authors:** Lin Xue, Danchen Han, Yingzi Wang

**Affiliations:** 1 School of Foreign Languages and Literature, Shandong University, Jinan 250100, China; l.xue@sdu.edu.cn; 2 College of Liberal Arts and Social Sciences, City University of Hong Kong, Hong Kong, China; danchehan3-c@my.cityu.edu.hk; 3 National Institute for Oriental Languages and Civilizations (INALCO), 75013 Paris, France

**Keywords:** identification, LOTE majors, ecological systems theory, higher education in China

## Abstract

This study investigates how Languages Other Than English (LOTE) students negotiate their identification amid growing uncertainties about the value of multilingual learning and language degrees. Drawing on ecological systems theory (EST), it identifies eight influencing factors ranging from microsystem to macrosystem that shape students’ identification. These factors are organised across three interconnected spheres: the individual sphere, shaped by personal interest and symbolic resonance with language and culture; the social sphere, driven by socially valued expectations in academic and career domains; and the relational sphere, grounded in interpersonal connections within the departmental community. Relational belonging—nurtured within inclusive and supportive peer–teacher networks—represents a key stabilising force, fostering students’ identification with the major. By proposing a dynamic model of identification, the study suggests that LOTE programmes should strengthen activities related to identity support and cultural engagement.

## 1. Introduction

Since the early twenty-first century, the humanities disciplines in higher education have faced increasing pressure due to the growing emphasis on career-oriented education and vocational training (Mintz, 2021; Morley, 2023; Troiani & Dutson, 2021). Within this context, language programmes have encountered a number of challenges, including declining enrolments, reduced institutional support, and growing public scepticism about their value (Ross, 2024). These pressures have been further compounded by the rapid development of generative artificial intelligence (AI), particularly large language models capable of performing efficient language translation, which has raised new uncertainties about the future of language education and language-related professions. These uncertainties are especially pronounced for students majoring in Languages Other Than English (LOTE), whose programmes, unlike English, are often perceived as having lower utility and limited career prospects (Dörnyei & Al-Hoorie, 2017; Y. Han et al., 2019). In this context, identification has become a key concept for understanding how LOTE majors make sense of their academic engagement and future aspirations.

Identification is defined as the process through which individuals integrate roles, values, and community expectations into their self-concept (Ashforth et al., 2008). As a form of psychological attachment, identification has been associated with lower dropout intentions, greater sustained engagement, and higher levels of satisfaction and motivation (e.g., Van Dick & Wagner, 2002; Weiß et al., 2023). Within higher education, identification has been examined across a range of disciplines. However, this body of research tends to concentrate on fields with clearly defined professional trajectories, such as health sciences, STEM (science, technology, engineering and mathematics), and teacher education, where the learning process is accompanied by a relatively clear future self-image. As a result, identification tends to be treated as a secondary construct, subordinate to identity as an institutionalised outcome of identification processes (Noble et al., 2014; Reissner & Armitage-Chan, 2024). For learners of LOTE, however, the university learning experience resembles a journey without a predetermined destination (Chan, 2016), making the process of identity construction more open-ended and uncertain.

Taking French majors in Chinese universities as the focal case, the present study draws on ecological systems theory (EST; Bronfenbrenner, 1979, 1986) to examine how LOTE students’ identification is shaped within the nested educational and sociocultural environments.

## 2. Literature Review

### 2.1. Conceptualisation of Identification

The concept of identification, referring to the psychological and social process through which individuals incorporate the values of a group into their self-concept (Johnson et al., 2006; Ashforth & Mael, 1989), has been developed across multiple theoretical traditions within the social sciences. First introduced by Freud, identification was initially conceptualised as an unconscious mechanism through which individuals internalise the traits of a lost loved one in order to maintain emotional attachment (Freud, 1917). Although grounded in a psychoanalytic thought, Freud later extended the concept from the parent–child relationship to larger social groupings, thereby highlighting its broader social dimension (Freud, 1922). Subsequent sociological perspectives further emphasised the interactive and socially constructed nature of identification. While psychoanalytic approaches largely conceptualised identification as a passive and defensive psychodynamic process (Laughlin, 1979), sociological theories increasingly portrayed it as a process through which individuals actively interpret and internalise social expectations through role-taking and social interaction (Mead, 1934; Parsons, 1991; Stryker, 1968).

The affective and social dimensions of identification were further developed in social identity theory and self-categorisation theory, in which identification has been viewed as a sense of belongingness to a particular social group (Tajfel & Turner, 2004; Mael & Ashforth, 1992; Ashforth & Mael, 1989). As Tajfel (1978) argued, social identity constitutes ‘that part of an individual’s self-concept which derives from his [or her] knowledge of his [or her] membership of a social group together with the value and emotional significance attached to that membership’ (p. 63). Building on this, Turner et al. (1987) introduced the notion of social identification to explain how individuals internalise group belongingness and integrate group values into their self-definition. Accordingly, identification can be understood as a tripartite process involving: (1) cognitive self-definition, (2) emotional belongingness to a group, and (3) normative alignment with its collective values (Tajfel, 1982; Ashforth & Mael, 1989). Later developments in identity control theory further emphasised the dynamic and negotiated nature of identification, conceptualising it as fluid, socially regulated, and continuously reconstructed through processes of identity verification and adjustment (Burke & Stets, 2009; Dashtipour, 2012; Rabbie et al., 1989).

### 2.2. Identification in Higher Education

Empirical research in higher education has demonstrated the significance of identification for individual outcomes. As a process central to identity formation, identification captures the ways in which students develop, negotiate, maintain, or withdraw attachment, alignment, and belonging to their academic field. In contrast, identity formation concerns the broader development of an evolving sense of self through the integration of knowledge, skills, experiences, values, aspirations, and role understanding over time (Fitzgerald, 2020; Reissner & Armitage-Chan, 2024; Trede et al., 2012). The two constructs are mutually constitutive: identification catalyses identity formation as individuals internalise collective values and expectations (Adams et al., 2006; Ashforth & Mael, 1989), while an established social identity shapes how subsequent identification is accepted, reinforced, or transformed (Ashforth et al., 2008).

Strong identification with academic majors is associated with lower dropout intentions (Burleson et al., 2021), higher levels of well-being (Toubassi et al., 2023), improved employability (Tomlinson & Jackson, 2021), greater career persistence (Hong, 2010), and long-term identity coherence (Lordly & MacLellan, 2012; Monrouxe, 2010). A range of factors have been found to strengthen identification, including future professional image (Keshmiri & Bahramnezhad, 2022), curriculum relevance (Kellar et al., 2023), and interactions with peers and instructors (Lordly & MacLellan, 2012; Walker et al., 2014). Recent work increasingly adopts a more contextual approach and pays attention to specific student groups (Hancock & Walsh, 2016; Park et al., 2018), reflecting an awareness of the situated nature of identification in higher education.

However, much of this literature is framed within broader discussions of *identity development* (Tannous et al., 2024) and focuses mainly on disciplines with clear and linear professional trajectories and institutionalised development pathways such as health, STEM fields, and teacher education (Chan, 2016). This focus tends to reinforce a normative view of identification as a rational and upward progression toward predefined roles (Reissner & Armitage-Chan, 2024). Yet identification is also an ongoing and sometimes ambivalent process through which students may both identify and disidentify with their field of study. The fluid and ambivalent aspects of students’ identification in many other disciplines remain underexplored.

### 2.3. Identification Among LOTE Majors in the Era of AI

Recent reviews of AI-assisted language learning have presented a largely positive picture, with most studies reporting beneficial effects on language achievement as well as affective and cognitive outcomes (Chang & Sun, 2024; Saarela et al., 2026; Y. Wang et al., 2026). However, parallel discussions beyond the language learning literature have raised concerns about the broader societal consequences of rapid AI development. In particular, some research has suggested that AI may substantially reduce demand for certain traditional language-related skills and occupations (Acemoglu et al., 2022). This contrast points to an important gap in the literature: relatively little attention has been paid to how AI influences students’ perceptions of the future value of language learning and language-related expertise.

This issue may be especially salient for language majors. Previous studies have often treated language majors simply as ‘language learners’ and grouped them with other students who study a foreign language as an elective (Caruso & Fraschini, 2021), a compulsory course (Zheng et al., 2019; T. Wang, 2023; Lanvers, 2016), or even a leisure activity. Building on this perspective, research has shown that language majors develop multiple and intersecting forms of identification, including learner identity (e.g., Teng, 2019), linguistic identity (e.g., Diao, 2017), cultural identity (e.g., Müller & Schmenk, 2016), gender identity (e.g., Bown et al., 2015), and national identity (e.g., Jackson, 2008). However, language majors differ from other language learners in important ways. They devote a considerable proportion of their university studies to intensive language learning and are expected to develop disciplinary expertise upon graduation. Their language learning is also closely tied to assessment, degree completion, and future career paths, making their identification processes complex and consequential.

These complexities may be particularly pronounced among students majoring in LOTE. Recent work has increasingly situated LOTE majors within broader institutional and sociocultural contexts. This literature suggests that motivation to learn LOTEs is often relatively fragile and shaped by sociocultural, institutional, familial and relational factors, which may contribute to fragmented or ambivalent forms of identities (Dörnyei & Al-Hoorie, 2017). Personal factors such as emotional engagement and multilingual orientation also play a role in shaping identity development and motivational trajectories (Dewaele et al., 2024; Wu & Forbes, 2025). Within these contextual and personal influences, comparisons between LOTEs and English are frequently highlighted (T. Wang & Liu, 2020; Z. Li, 2025). While LOTE learning is often framed as an investment associated with career development, advantages associated with multilingual competence, and future opportunities (Xue, 2022; Göksu & Louis, 2025), some studies have argued that LOTE education policies may paradoxically reinforce the dominance of English rather than promote multilingual diversity (Lu & Shen, 2022). Taken together, this body of work has mainly approached LOTE majors from the perspective of motivation, investment, and identity development, highlighting their relatively vulnerable position within broader educational and sociolinguistic structures. However, less attention has been given to LOTE majors’ sense of disciplinary belonging, particularly how they identify with or disidentify from their academic major under increasingly uncertain sociolinguistic and educational conditions.

### 2.4. An EST-Based Multilevel Framework

As a developmental and contextually embedded process, identification is shaped by the contextual conditions in which learners are situated. Prior research has explored relevant social and discursive influences, such as peer interaction, institutional discourse, and sociocultural narratives (e.g., Kellar et al., 2023; Noble et al., 2014). However, these studies often treat such factors separately, without adopting a holistic perspective that captures how multiple, nested environmental systems operate together.

EST (Bronfenbrenner, 1979, 1986) provides a multilevel framework for addressing this limitation. It locates identification within layered, nested environmental systems, including the microsystem (e.g., peer and teacher relationships), mesosystem (e.g., connections between academic and familial settings), exosystem (e.g., institutional policies), macrosystem (e.g., cultural discourses), and chronosystem (e.g., temporal shifts across the other ecological systems) (Bronfenbrenner, 1979, 1986; Krishna et al., 2024; Ozaki et al., 2020). This framework has been used to examine how identification is shaped through interactions across multiple educational and social contexts (Hua et al., 2022; Krishna et al., 2024; Ozaki et al., 2020). Drawing on EST, this study examines how LOTE majors in Chinese universities construct their identification within changing and uncertain academic and career landscapes. It addresses the following questions:

(1) What contextual factors influence LOTE majors’ identification?
(2) How are these influences mediated and negotiated through interactions across ecological systems?

## 3. Methodology

To answer the research questions, a qualitative multi-case study approach was adopted. Qualitative research emphasises processes rather than outcomes (Bogdan & Biklen, 2006), making it well suited to the study of dynamic phenomena such as identification. In addition, the case study approach enables an in-depth understanding of complex phenomena and facilitates close examination of the contextual factors shaping participants’ experiences (Merriam, 1988). This approach is particularly appropriate for the present study, given its focus on LOTE majors’ identification processes as situated within specific contextual conditions.

### 3.1. Participants and Context

All of the participants were multilingual learners who had studied English in secondary school and later pursued a LOTE as their university major. French was selected as the focal foreign language programme due to its global significance and institutional prominence. It is the second most widely learned foreign language worldwide, with over 300 million speakers (Organisation Internationale de la Francophonie, 2022). In China, French is among the most commonly offered foreign language majors in higher education, available at approximately 160 universities (Ministry of Education of the People’s Republic of China, 2024). Its institutional presence has further expanded in the context of national strategies such as the ‘Belt and Road’ Initiative, which emphasises multilingual and multicultural competencies. This combination of global prominence and local embeddedness makes French a suitable case for examining LOTE students’ identification.

Ten final-year French majors were recruited through maximum variation sampling (Patton, 2002). Participant selection was guided by variation in gender, academic background, and postgraduation orientation to capture a broad spectrum of identification experiences (Table 1). In accordance with ethical guidelines, the participants were fully informed of the study’s aims and procedures, and the research began only after obtaining their voluntary consent. All personal data were kept confidential, and the participants were treated without judgement or bias.

### 3.2. Data Collection and Analysis

Data were collected between February 2024 and March 2025 through semi-structured interviews (I), autobiographical narratives (AN), and multiple rounds of longitudinal follow-up interviews (FI). The initial one-on-one interviews (lasting for 1–3 h) were guided by an EST-based interview protocol. All interviews were audio-recorded with the participants’ consent and transcribed verbatim. Participants were then invited to submit voluntary autobiographical narratives reflecting on their experiences as French majors. This sequence was designed to reduce researcher bias, support deeper reflection, and prompt recollection of previously unnoticed experiences (Weiss, 1995). Follow-up interviews were conducted iteratively to clarify ambiguities, validate preliminary interpretations, further explore emerging themes, and track evolving perceptions over time. The final dataset included over 210,000 Chinese characters from the initial interviews, approximately 10,000 from the narratives, and around 5000 from the follow-up interviews.

The data were analysed using NVivo 14, following a three-stage coding procedure that combined inductive and deductive approaches. The deductive approach was informed by EST, while the inductive approach allowed codes to emerge from the data. The analysis began with repeated readings of the interview transcripts to achieve familiarity with the dataset. Open coding was conducted inductively to identify recurring concepts and agents (e.g., ‘feeling supported by peers’, ‘comparison with top students’). Given the study’s focus on identification, in this stage, particular attention was paid to the participants’ expressions of attachment, alignment, belonging, commitment, and distancing in relation to their academic major. These initial codes were then grouped into axial categories; for instance, peer support, peer competition, and peer atmosphere were combined under the label ‘peer’. The resulting axial codes were refined until they demonstrated internal homogeneity and external heterogeneity and adequately captured patterns across the dataset (Braun & Clarke, 2006). Axial codes were then mapped deductively onto the five ecological layers: microsystem, mesosystem, exosystem, macrosystem, and chronosystem. The allocation of codes across ecological levels was informed by both previous literature and the relative criteria of contextual scale and proximity. For example, employment prospects and social discourse were classified as part of the exosystem, drawing on D. Liu et al. (2022) and Eriksson et al. (2018). Macrosystem-level factors were distinguished from exosystem-level factors by their broader scale of influence, capturing global or cross-national forces rather than influences more directly embedded in the Chinese context.

To enhance the credibility and trustworthiness of the analysis, the research team engaged in ongoing discussions throughout the coding process. In addition, a second researcher independently coded approximately 30% of the dataset (the data obtained from Jordan, Emma, and Alba). Coding comparisons were conducted across different stages of analysis. Where differences emerged, the researchers discussed not only the coding decisions themselves but also how the codes should be interpreted and defined. The axial coding stage generated the most discussion, particularly regarding category boundaries and decisions on whether categories should be created, merged, or separated. Coding discrepancies were discussed until a consensus was reached. Methodological triangulation was conducted by comparing evidence across interviews, autobiographical narratives, and follow-up interviews. Where inconsistencies emerged, the participants were invited to clarify their accounts in follow-up interviews, allowing the research team to verify interpretations and refine emerging codes. The participants also reviewed and validated the transcripts and interpretations.

All of the data were coded in Chinese, the native language shared by the participants and the researchers, to preserve contextual meanings and minimise the loss of nuance during analysis. All of the excerpts presented in this article were subsequently translated into English. The first and third authors collaboratively reviewed the translations to ensure their accuracy and fidelity to the original meanings.

### 3.3. Researcher Positionality

The research team brought both insider and outsider perspectives to the study. The first author is a professor in a French-major programme, while the third author is a former French-major student. Their familiarity with the field provided valuable contextual knowledge and facilitated a nuanced interpretation of the participants’ experiences. In contrast, the second author approached the study from a more external position, contributing critical distance and alternative perspectives to the data analysis. Regular discussions among the research team throughout the coding and interpretation processes enabled assumptions to be questioned and analytical decisions to be critically examined. This combination of insider understanding and outsider scrutiny increased the reflexivity of the research process and supported a balanced interpretation of the data.

## 4. Findings

Based on the data analysis, the following section presents the key factors shaping participants’ identification and examines how these factors influence this process. Although the contextual influences were dynamically interconnected, the analysis will address each factor individually to allow for a more in-depth exploration. Temporal changes (i.e., the chronosystem) are discussed within the microsystem, exosystem, and macrosystem in which they were identified.

While all ecological systems informed the analysis, the participants’ accounts were concentrated primarily within the microsystem, exosystem, and macrosystem, with limited evidence relating to mesosystem processes. The mesosystem encompasses the interrelations between two or more settings in which an individual actively participates (Bronfenbrenner, 1979). However, the participants’ accounts were situated predominantly within the university context, potentially due to the characteristics of the participant group: as adult students, they reported relatively limited interaction between family and university settings. Furthermore, compulsory internships were generally not a requirement of the French degree programme, resulting in relatively few opportunities for interaction between university and workplace contexts.

### 4.1. Microsystem

#### 4.1.1. Teachers: Academic Guides, Emotional Anchors and Cultural Ambassadors

Teachers played a central role in shaping the participants’ identification, particularly through their disciplinary expertise and academic guidance. For instance, Calandra (I) described her initial anxiety about the rapid advancement of artificial intelligence (see also Section 4.3.1), fearing that translators might be rendered obsolete by machines. However, her teacher’s perspective reframed this concern: ‘What we need to do is not fight against ChatGPT or resist it but learn to coexist with it and turn it into something that benefits us’.

Beyond their professional guidance, the teachers served as emotional anchors. Nine participants described feeling ‘seen’ and valued through classroom interactions and informal exchanges with teachers. Cécilia (FI), for example, recounted how being ‘noticed by her teacher’ during class made her ‘hesitate in [her] decision about whether to switch majors’. Similarly, Jordan (I) highlighted how his teacher’s encouragement helped him maintain a peaceful attitude towards French studies, even in the face of fluctuations in academic performance: ‘If you encounter a teacher who only values grades, you may become more easily bored or develop a more extreme attitude toward French. But our teacher is so nice. He supports us to pursue what we want to do. […] He believes that your success is defined not by him but by you’.

In addition, the teachers were credited with fostering an inclusive departmental atmosphere. Many of the participants linked this inclusivity to teachers’ openness, which they saw, in many cases, as influenced by their exposure to French culture (see also Section 4.3.2). Maity (FI) described the French department as ‘a space where everyone respects differences and expresses themselves freely’, attributing this to teachers’ overseas experiences. Alba (I) added, ‘French thought is particularly egalitarian, and our teachers somewhat reflect this in all of their courses… It’s definitely not just about teachers imparting knowledge’. Charlotte (I) reinforced this idea by contrasting the teaching styles in French and other departments, emphasising that the former appeared more freedom-oriented and encouraged students to explore their personal interests:

‘My teachers have all studied in France, and I also think that French major teachers have a different mindset from teachers in [other programmes]. They seem freer and allow me to find my own meaning in life…’

Thus, teachers served not only as academic and emotional anchors but also as cultural ambassadors, helping students navigate the professional and cultural significance of studying French. However, their influence was not uniformly enabling. Vanille (AN) recalled being publicly criticised by a teacher for prioritising extracurricular activities over academic study. While she acknowledged the teacher’s good intentions, the experience exerted a lasting impact on her long-term career aspirations: ‘In my mind, this teacher has been marked with a big cross… I thought, if pursuing an academic path means I must go through his guidance, then I would rather give up on this path’.

#### 4.1.2. Peer Interaction: Community Bonds and Comparative Benchmarks

Peer interaction was another key microsystem factor shaping the participants’ identification. Nearly all of the participants mentioned peer interaction as a critical factor contributing to shifts in their attitudes towards or feelings about their French studies. These relationships served as both emotional support and reference points for self-evaluation.

SYan (I), who had not chosen the major voluntarily, attributed her continuation partly to peer support. She further emphasised the broader importance of emotional support provided by her peer network: ‘During a period of early adulthood, when you’re starting to face many things on your own, being in an environment where people around you support, care for, and protect you is really important. I value this a lot’.

Such positive peer relationships contributed to a sense of collective identity. Maity (FI) underscored how camaraderie strengthened his identification, suggesting that the French department attracted like-minded people: ‘I feel that foreign language majors are more likely to attract people who resonate with me… If I were placed in a physics or chemistry programme, I would probably find it difficult to make such good friends’.

However, peer comparison sometimes generated feelings of inadequacy. Charles (FI) experienced lower self-evaluations when outperformed by classmates with prior French exposure:

‘I felt an unprecedented sense of confusion and regret, questioning why I had applied to XX University, why I had chosen a foreign language major, why I had come to XX campus, and why I hadn’t changed my major. […] Because at that time, the two bottom students transferred, leaving only me and two other boys in the class… It’s easy for them to learn, but difficult for me. […] And I heard that those who transferred are doing well, while I still felt like a mess’.

Charles described the emotional turmoil triggered by witnessing classmates’ stronger language abilities, which led him to question his own aptitude and decision to remain in the major. In contrast, for Vanille (I), observing peers’ disillusionment reinforced her identification. She reflected: ‘The thing about “liking” … it… [Hesitation] Well, I don’t really know. […] I can only say that the difference between me and the others is that a lot of friends around me found out they’d chosen the wrong major after four years of study and didn’t like learning languages at all. Me, I still like it a lot. Yes, that’s how it is’.

#### 4.1.3. Curriculum Design: Room for Growth, Yet Limited in Professional Reach

Curriculum design played a dual role in the participants’ accounts: while some criticised the curriculum’s focus on language skills, others valued the flexibility it offered for personal growth.

A frequently reported concern was the curriculum’s overemphasis on language acquisition and standardised testing, which narrowed perceived career options. As Jordan (I) noted, ‘If we aim to find jobs that are well-matched, the options are mostly confined to translators or language teachers. […] Therefore, the professional scope of the programme is quite restricted’. This perception has been strengthened by the development of AI, as translation is increasingly seen as one of the jobs most likely to be automated (see Section 4.2.1 and Section 4.3.1).

This language-centred training also left students underprepared in broader academic skills. Vanille (FI), who pursued graduate studies, reflected: ‘During our undergrad studies, a lot of time was spent on pure language learning. […] There was a lack of training in professional applications, such as writing academic papers’.

Some of the participants also observed that the emphasis on language training did not always translate into effective real-world communication. Recalling his early days in France, Maity (FI) shared: ‘During my first week in France, I felt like I had lost the ability to speak. It felt as if I had learned nothing in my four years of study, and I was embarrassed to tell others I had studied French for four years’.

At the same time, several participants highlighted positive, humanistic aspects of the curriculum. The relatively light workload was seen as allowing time for emotional balance and personal development, particularly in comparison with more intensive programmes. Maity shared that the manageable workload enabled him to enjoy a more balanced university life: ‘I imagined what it would be like if I were in a law or science program—I would probably be much more exhausted. I don’t want such a tiring university life’.

The participants also valued the curriculum’s thematic diversity: ‘covering translation, interpretation, journalism, literature, and even some economics’ (Alba—I). Calandra further emphasised how specific elements of the curriculum, such as exposés (oral presentations in French), helped to build her confidence and promoted her personal and professional development: ‘Doing exposés really helps you grow. After four years of practice, I feel very confident speaking in front of an audience. And my ability to control a room has significantly improved’.

### 4.2. Exosystem

#### 4.2.1. Employment Prospects: Constraints Locally, Incentives Globally

Employment prospects played a significant role in shaping the participants’ identification. Five participants reported choosing LOTE programmes because they believed that this choice offered a competitive edge. Charles (I) considered language skills ‘a real asset’, distinguishing him from other humanities majors. Charlotte (I) echoed this view, recalling how teacher advice influenced her decision-making: ‘I wanted to switch to finance, but my teacher made a good point: if I spent my university years mastering French, studying finance later would be much easier—but not the other way around’.

However, this initial optimism often gave way to disillusionment as students encountered the scarcity of direct job opportunities in French-related fields. In her autobiographical narrative, Vanille reflected on the gap between initial expectations and reality: ‘Like many others, I started with the dream of working at the Ministry of Foreign Affairs or becoming a top translator. But in reality, only a few make it that far’. Emma (I), who was actively job hunting at the time, explicitly attributed her job-hunting difficulties to what she perceived to be the limited scope of the programme: ‘Because of the limitations of the French major, I discovered that there are very few jobs that completely match’. Even the job widely regarded as a ‘match’ for French majors—translation—was perceived as increasingly threatened by recent developments in AI (see also Section 4.3.1), as ‘AI can already handle most routine translation tasks’ (Emma—I).

Interestingly, French-speaking regions in Africa were frequently seen as a potential labour market for French graduates, unlike graduates of other LOTE majors, offering both demand and financial return. Charlotte (I) viewed this as a potential stepping-stone: ‘You could work in Africa for a few years, earn money, and then use those earnings to study in France or pursue other opportunities’.

However, most of the participants associated the African continent with underdevelopment, instability, and disease, leading them to ultimately decide against this option. Calandra (I), for instance, acknowledged the risks associated with high-paying jobs in Africa: ‘Working in Africa pays a lot, but that money comes with risks. For example, there’s malaria, and life is inconvenient’. Charles (I), although initially interested, also stepped back after reflection: ‘I thought, earning money is for the sake of life with family, so I’d rather stay in China’.

#### 4.2.2. Social Discourse About LOTE Programmes: A Perceived Hurdle

If the limited availability of closely matched employment opportunities for LOTE graduates—highlighted in both prior research (Y. Han et al., 2019) and participants’ accounts—had already unsettled many students, the prevalent social discourse in China further intensified their uncertainty.

The belief that LOTE majors lead to a professional ‘dead-end’^1^—a term repeated by almost all participants—was widely internalised. Vanille (I) described her shock upon encountering an online community that strongly discouraged LOTE studies: ‘Even postgraduates from XU and YU^2^ are still being discouraged. I wondered, is it really that uncertain? Oh my God!’ Cécilia (I) similarly recalled social media discussions in which students expressing interest in LOTE majors were met with comments such as ‘Run!’, accompanied by claims that ‘there is no job that fits.’ These narratives were further reinforced in relation to the development of AI, as noted by Cécilia: ‘In Douyin^3^ comment sections, people often say that studying foreign languages will inevitably lead to unemployment, mentioning tools like iFlytek translators^4^ and ChatGPT’.

However, not all participants accepted this narrative. Maity (I) challenged the generalisation: ‘Saying it’s hard to find a job with a French degree is based on the majority of cases but leads to a general conclusion. (…) I’m not very anxious about this’.

Calandra’s experience, marked by a clear shift, aligned with Maity’s perspective that personal conditions can differ from general trends and individual agency plays a decisive role. During her senior-year job search, she admitted, ‘I was often wondering, did I choose the wrong major?’ (Calandra—I). Over time, however, her doubts gave way to a more proactive viewpoint with an emphasis on personal agency: ‘Not finding a job is relative. Some people succeed. It depends on personal effort’ (Calandra—FI).

#### 4.2.3. Postgraduate Recommendation Policy: A Powerful Motivator

Although mentioned less frequently, China’s postgraduate recommendation policy exerted an intriguing influence on the participants’ identification. This policy allows high-achieving undergraduates to bypass the highly competitive national entrance examination and gain direct admission to master’s programmes. Despite being perceived as a lower-risk pathway, the process remains highly selective and is commonly associated with the image of being a ‘good student’ (Y. Zhang & Huang, 2022).

Cécilia’s experience illustrates the strategic role of this policy. Although she initially considered transferring to the English major for better career prospects, she began to hesitate as the deadline for changing majors approached at the end of her first year. She linked her hesitation directly to the recommendation policy: ‘As I had ranked first in my class, I thought I could secure a recommendation for graduate school in the future. I felt that if I switched, I might not get the recommendation’ (Cécilia—I). Ultimately, Cécilia remained in the French department and was later admitted to a master’s programme in French language and literature through departmental recommendation. Reflecting on the experience, she directly acknowledged how crucial the policy had been in shaping her identification: ‘If I hadn’t secured the recommendation, as I would not have had the energy to take the national examination, I would have just graduated and gone to work. I’d have regretted studying French, feeling like I’d wasted four years without learning much’ (Cécilia—FI).

For Cécilia, postgraduate admission appeared to hold greater significance than disciplinary commitment itself. The French major functioned as a strategic pathway through which she could benefit from the recommendation policy. Despite ongoing reservations about the field, she continued into a master’s programme in French language and literature, acknowledging the difficulty of switching disciplines within the recommendation system.

Nonetheless, the policy does not exert a universal influence. Charlotte (I), despite being eligible, had different priorities: ‘At that time, I was already determined to go abroad, so I never even considered that path’. She applied to French business schools instead, aligning her choice with her long-term career goal.

### 4.3. Macrosystem

#### 4.3.1. Advancements in AI: A Formidable Challenge

Recent advancements in AI have introduced new challenges to language majors’ identification, especially in relation to translation, the field most associated with French studies.

Participants recognised the linguistic and cultural capacities of AI, which some considered ‘almost unmatched by undergraduates’ (Cécilia—FI). As discussed in the Section 4.2.1, this raised concerns that job opportunities for French majors might be further reduced: ‘At the rate AI is developing, it will soon replace a large portion of human translators’ (Maity—I). At the same time, the participants believed that AI would never fully replace human involvement in all aspects of translation. Calandra (I), for example, emphasised that the interpersonal and situational demands of interpretation are beyond AI’s comprehension: ‘Unexpected situations definitely require human communication. […] For more advanced levels, it is really hard, very hard for AI to replace human translators’.

The tension between AI’s power to ‘not replace everyone’ and its potential to ‘replace a large portion of people’ produced divergent effects on the participants’ identification. For some, like Emma, AI reinforced existing pessimism: ‘A few years ago, studying foreign languages was a good choice, but now I don’t think so. AI can already handle most routine translation tasks’ (Emma—I). Calandra’s perspective, however, was entirely different: although she had initially perceived AI’s development as a significant threat to language majors, she had ultimately come to see herself as part of the group that would not be replaced by AI:

Interviewer: So, you believe that AI will not replace good translators, and you will become one of those translators?

Calandra: Yes! Of course! (Calandra—FI)

While the impact of AI on language majors was widely acknowledged, it was rarely a primary cause of disidentification among the participants. Most of the participants had decided against translation-related careers even before the recent development of AI, mainly due to labour market constraints or personal interests. In this sense, AI functioned mainly as a reinforcing factor: for many, it confirmed their existing disidentification; for a few, like Calandra, it clarified a more selective, expert-oriented translator identity.

#### 4.3.2. Foreign Culture: Initial Motivation and Sustained Drive

In contrast with the more ambivalent factors discussed above, French culture consistently exerted a positive influence on the participants’ identification. It not only served as the initial motivation for choosing the major but continued to be a sustained source of engagement and emotional connection throughout their university experience.

For many, cultural interest was an initial driver for choosing the major. They described a strong affinity with perceived French values such as introspection, freedom, and aesthetic sensibility, as shared by Maity (I): ‘The French literature and philosophy I encountered in high school resonated with my introspective self, and therefore I felt a sense of closeness and anticipation for the whole French culture’. Similarly, Calandra (I) described being attracted by the popular perception that ‘French is the most romantic language’, explaining how cultural imagery and affective symbolism had contributed to her early enthusiasm.

This initial attraction was often reinforced and further developed through academic study and intercultural experiences. Charlotte (I), recalling her exchange experience in France, noted:

‘Although things moved slowly, everything was progressing in an orderly manner. In contrast, I was constantly rushing from one thing to another, which felt meaningless and only made me more anxious. […] That sense of pacing gave me a chance to slow down and re-centre myself’.

The positive influence of French culture on the participants’ identification was also evident in their descriptions of the personal changes shaped by exposure to French culture. For instance, Vanille (AN) shared that despite being a long-time ‘good student’, she had once chosen to skip class to participate in a student association activity. She reflected: ‘Perhaps it was my exposure to the ideas about freedom and self-exploration in French culture. This contributed to a shift from my previously conformist nature, which I really appreciate’.

Overall, French culture played a continuous role throughout the participants’ university experience, operating on multiple dimensions. The initial attraction to French culture served as a primary motivator for major selection, while sustained cultural engagement during their studies not only met but also often exceeded the participants’ expectations. This was evidenced by their expressed satisfaction with both the academic experience and the accompanying personal transformation.

## 5. Discussion

Drawing on EST and the concept of identification, this study identified multiple context-specific and era-related factors across the microsystem, exosystem, and macrosystem that shape the identification of French majors at Chinese universities. The findings indicate that participants’ identification processes were continuously negotiated through interactions across multiple ecological levels. In this sense, participants’ identification emerged as fluid, fragmented, and at times contested under increasingly uncertain sociolinguistic and educational conditions.

### 5.1. Bidirectional Ecological Interactions and Negotiated Identification

Consistent with previous ecological research, the findings show that proximal systems exert a strong influence on students’ identification processes due to their immediacy and emotional significance (Bronfenbrenner & Morris, 2006; Hayes et al., 2017). Teachers and peers directly shaped students’ academic and emotional experiences, while also mediating the influence of broader institutional, technological, and sociolinguistic conditions. In this sense, exosystem- and macrosystem-level influences were often interpreted and enacted through everyday interactions within proximal educational settings (El Zaatari & Maalouf, 2022; McHale et al., 2009). These findings also resonate with The Douglas Fir Group’s (2016) multilayered framework of language learning and identity, which views language-related experiences as emerging through interactions of individual, relational, institutional, and ideological dimensions. For example, teachers and peers functioned not only as sources of emotional support but also as carriers of French cultural values and disciplinary meanings. Similarly, the influence of AI at the macrosystem level permeated multiple layers of context. It heightened the participants’ perceptions of employment difficulties in the exosystem, which led to increased questioning of the curriculum structure in the microsystem. In this sense, this study contributes to the literature on AI-assisted language learning. While previous studies have largely highlighted the positive educational benefits of generative AI (e.g., M. Liu & Reinders, 2025), the present study suggests a more complex picture in relation to disciplinary identification. Rather than uniformly enhancing engagement, AI prompted some of the participants to question the value and future relevance of their field of study, while reinforcing commitment among others. This finding echoes recent calls for a more tempered perspective on the educational implications of AI technologies (Y. Wang et al., 2026).

However, our findings also extend existing ecological accounts by illustrating that proximal systems do not simply transmit macro-level influences in a top-down manner. Rather, teachers and peers actively interpret, negotiate, and at times resist broader societal discourses. This supports recent calls for greater attention to the dynamic interplay between person, process, and context in language education research (Chong et al., 2023; Tong & An, 2024). Calandra’s case illustrates this negotiation process clearly. Her initial anxiety about AI and the future value of French was not reduced through engagement with AI itself but through her teacher’s reframing of disciplinary meaning, which strengthened her confidence in the major. Similarly, the participants’ tendency to associate Africa with underdevelopment and disease may have stemmed partly from curricular representations that privilege metropolitan France over other Francophone contexts, particularly Africa (H. Zhang et al., 2024). These selective representations may have narrowed the students’ cultural understanding of the diversity of the Francophone world and limited their engagement with a broader range of linguistic, cultural, and professional possibilities. Taken together, the findings suggest that ecological influences are not linear or unidirectional but continuously negotiated within proximal educational relationships.

### 5.2. Fragmented Identification Under Social and Individual Expectations

Beyond these interconnected influences, participants’ identification was also shaped by ongoing tensions between socially driven expectations and individual aspirations. Socially oriented identification was closely associated with external expectations and dominant societal norms in China, including peer comparison, employment prospects, social discourse, postgraduate recommendation policies, and increasing concerns surrounding AI and the future value of LOTE degrees. In contrast, individually oriented identification was linked to intrinsic motivation, emotional attachment to the French language and culture, and supportive relationships with teachers and peers. This tension is consistent with perspectives that view identity as emerging from interactions between individual dispositions and sociocultural environments (Tanti et al., 2008; Nasiri & Shokrpour, 2024), as well as distinctions between personal and social dimensions of the self (Sedikides & Brewer, 2001). However, these dimensions did not function as clearly separated categories. Rather, they overlapped and were continuously negotiated within broader sociolinguistic and educational contexts.

When the two orientations were relatively aligned, participants tended to develop more stable and coherent forms of identification (Sedikides & Brewer, 2001). This was evident among participants such as SYan, Charlotte, Alba, Calandra, and Maity, who managed to balance personal interest with external expectations. Some achieved this balance through postgraduate transitions that responded to employability demands while maintaining emotional attachment to French. Others maintained relatively stable identification through sustained engagement with French-related academic or professional pathways.

More commonly, however, identification was experienced as fragmented, unstable, and internally contested. Emotional attachment to French often exerted a strong ideological and affective pull, even in the absence of clear professional or economic returns (Oakes, 2013; Palmieri, 2018), with several participants describing French language and culture as central to their worldview and personal values. At the same time, identification with the French major was frequently weakened by socially valued expectations related to academic achievement, employability, and upward mobility. This finding aligns with previous research on LOTE motivation, which shows that learners’ ought-to selves play an important role in shaping major choice, investment, and learning behaviour (Lanvers, 2017; Leal Brancher & Yun, 2025).

Uncertainty was further intensified by widespread narratives surrounding AI and the perceived declining value of French in the labour market. In this context, maintaining the image of a ‘successful’ or ‘promising’ student often became more important than sustaining long-term disciplinary commitment. Some participants, therefore, strategically engaged with postgraduate recommendation systems to retain symbolic academic value and external recognition. For Cécilia and Vanille, for example, continuing in French functioned less as a sign of stable disciplinary identification than as a way of maintaining their socially recognised status as high-achieving students (Lai & Jung, 2025). This phenomenon is consistent with previous research showing that academic achievement remains a key marker of social value in China and other East Asian contexts (Huang & Gove, 2015; Tao & Hong, 2014). Peer comparison, while sometimes perceived as motivating (C. Ma & Chen, 2024), more often intensified self-doubt and disciplinary insecurity, particularly among students who viewed themselves as less competitive or less committed (C. Liu et al., 2024). These findings reflect the persistent influence of exam-oriented and rank-based educational structures on students’ academic self-concepts and future-oriented decisions (Z. Wang, 2023).

Importantly, the findings suggest that participants’ identification rarely took the form of either full identification or complete disidentification. Rather, participants continuously negotiated between emotional attachment to French and socially recognised achievement under increasingly uncertain educational and sociolinguistic conditions.

### 5.3. Relational Belonging as a Source of Identification Stability

Another key finding concerns the stabilising role of relational belonging within the microsystem. While previous studies have often treated teacher support and peer influence as separate factors (Maunder, 2018; Meehan & Howells, 2019), the present study highlights their combined influence as part of an interconnected relational ecology. Participants’ identification was strengthened not simply through individual encouragement, but through sustained experiences of emotional safety, mutual recognition, and collective belonging within teacher–peer communities. For some participants, identification with the French major was maintained less because of its perceived instrumental value than through emotionally meaningful relationships within the departmental community.

Relational processes thus functioned as an ecological buffer against broader pressures toward disidentification, including employment anxiety, sociolinguistic marginalisation, and uncertainty about the future value of LOTEs. For participants such as Maity and SYan, relationships with teachers and peers provided not only academic support but also emotional validation and a sense of disciplinary belonging. These findings resonate with previous research suggesting that identity formation is closely connected to proximal interpersonal relationships, through which individuals develop self-understanding, emotional security, and motivational orientation (Higgins & May, 2001). Higgins and May (2001) further argued that self-guides derived from significant others can either support or destabilise identity development depending on their alignment with individuals’ own perceptions and aspirations. The present findings extend this view by suggesting that relational belonging can, in some cases, exert a more immediate influence on identification than disciplinary interest or career considerations.

This relational dimension may be particularly relevant in East Asian educational contexts, where identity formation is often closely linked to interpersonal relations and collective recognition rather than individual autonomy alone (Cross & Morris, 2003). Under increasing uncertainty about the value of LOTEs, emotionally supportive relationships with teachers and peers therefore became an important source of identification stability. More broadly, the findings suggest that identification in this study should not be understood solely as an individual cognitive process, but as a relationally mediated and ecologically situated experience. By foregrounding relational belonging within proximal educational contexts, this study contributes to emerging discussions of identification as fluid, negotiated, and socially embedded within broader educational and sociolinguistic contexts.

**Figure 1:**
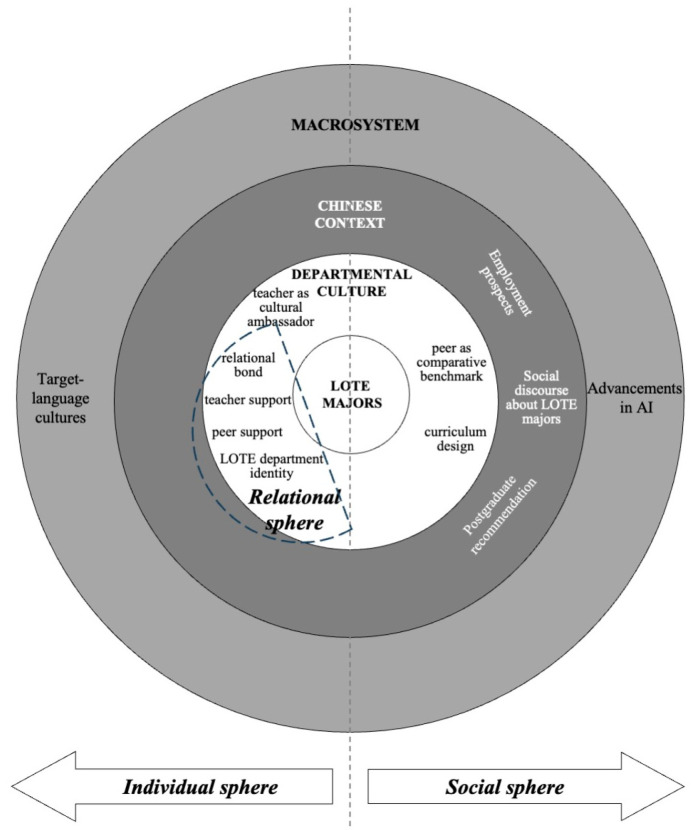
An ecological model of LOTE majors’ identification.

Figure 1 summarises the multilevel and multidimensional factors shaping LOTE majors’ identification. LOTE majors’ identification was shaped by interconnected factors operating across the departmental context, the Chinese context, and the broader cross-national context. As illustrated in the figure, these factors can be grouped into three spheres: individual, social, and relational.

## 6. Conclusions

Taking French majors as the focal case, this study examined how LOTE majors in Chinese universities construct their identification, revealing it as a dynamic and multilayered process shaped by the interaction of the individual, social, and relational spheres. The socially oriented identification was linked to external pressures, including academic competition, career concerns, and AI disruption. The individually oriented identification was grounded in intrinsic motivation and alignment with French cultural values. The relationally oriented identification, formed through sustained teacher–peer networks, emerged as a key stabilising force that helped reinterpret external pressures and support disciplinary commitment. Across these processes, AI operated as a cross-layer influence that permeated these spheres, functioning alongside other sources of uncertainty and reinforcing existing concerns regarding employability and future opportunities. The findings extend existing frameworks by highlighting the bidirectional nature of ecological influence. Rather than functioning as passive recipients of macro-level forces, students and their proximal environments actively interpret, negotiate, and sometimes resist these pressures.

This study is not without limitations. First, the sample was limited to ten French majors in Chinese universities. Therefore, the findings should be interpreted with appropriate contextual caution. Rather than seeking statistical generalisability, the study aims to provide contextually grounded insights that may offer analytical relevance to similar LOTE programmes and educational settings. A larger and more diverse sample, including students from other language majors and different cultural contexts, would yield a more comprehensive understanding of identification in uncertain times. Second, the data collection period (one year) may not have fully captured long-term changes in identification. Future longitudinal studies are needed to examine developmental trajectories in greater depth.

Although rooted in the Chinese context, this study offers context-sensitive suggestions that may resonate with language programmes facing similar pressures, particularly in the era of AI. At the microsystem level, the findings point to the value of identity-oriented pedagogies in language education that go beyond a focus on skills training. Practices such as peer mentoring and student-led activities can foster academic belonging and purpose (Chaffee et al., 2025). Where appropriate, curricular reforms could also be considered to diversify cultural content and reduce reliance on dominant-culture narratives. At the exosystem level, career-oriented training models could be developed to align with students’ future employment opportunities. The recent emergence of ‘foreign language +’ programmes in China (Y. Li & Chen, 2025)—offering dual degrees such as French + Business or Spanish + Language Education—reflects a trend toward cultivating interdisciplinary talents by integrating humanistic interests with adaptable career pathways. At the same time, participants frequently rely on online sources for career information, which may be biassed or misleading and can contribute to anxiety. This highlights the need for clearer institutional career guidance. At the macrosystem level, institutions may also draw on the cultural appeal of foreign languages and frame AI as an empowering tool rather than a threat (Y. Ma & Chen, 2024). However, maintaining a balance between affective engagement and instrumental goals in language learning remains a continuing challenge for both policy and pedagogy.

## Figures and Tables

**Table 1:** Participants’ background information.

Name	Gender	Academic Background	Post-Graduation Plans
Cécilia	F	Ordinary Comprehensive University	MA in French Language and Literature (China)
Maity	M	Ordinary Comprehensive University	MA in Linguistics (France)
SYan	F	Top-tier Science and Engineering University	Juris Master (China)
Vanille	F	Ordinary Foreign Language University	MA in French Language and Literature (China)
Charlotte	F	Ordinary Finance and Economics University	Master in Management (France)
Charles	M	Top-tier Comprehensive University	Employment
Jordan	M	Top-tier Foreign Language University	Master in Management (France)
Emma	F	Specialised Foreign Language College	Employment
Alba	F	Ordinary Foreign Language University	Employment
Calandra	F	Ordinary Foreign Language University	Employment

## Data Availability

The datasets generated and analysed during the current study are not publicly available because the participants did not provide explicit consent for open data sharing beyond the current project. Subject to approval by the relevant institutional ethics authorities and with the participants’ consent, anonymised data may be made available from the corresponding author upon reasonable request.

## Notes

1 ‘Dead-end’ is a commonly used (humorous) term among Chinese university students, describing certain academic fields with uncertain prospects, like a deep pit with no bottom in sight.

2 Top foreign language universities in China.

3 A Chinese short-video platform.

4 A well-known Chinese translation tool.

## Abbreviations

The following abbreviations are used in this manuscript:

LOTE	Languages Other Than English
AI	Artificial Intelligence
STEM	Sciences, Technology, Engineering and Mathematics
EST	Ecological Systems Theory
